# Multicolor T‐Ray Imaging Using Multispectral Metamaterials

**DOI:** 10.1002/advs.201700982

**Published:** 2018-03-25

**Authors:** Zhitao Zhou, Tao Zhou, Shaoqing Zhang, Zhifeng Shi, Ying Chen, Wenjian Wan, Xinxin Li, Xinzhong Chen, Stephanie N. Gilbert Corder, Zhanglong Fu, Liang Chen, Ying Mao, Juncheng Cao, Fiorenzo G. Omenetto, Mengkun Liu, Hua Li, Tiger H. Tao

**Affiliations:** ^1^ State Key Laboratory of Transducer Technology Shanghai Institute of Microsystem and Information Technology Chinese Academy of Sciences Shanghai 200050 China; ^2^ School of Graduate Study University of Chinese Academy of Sciences Beijing 100049 China; ^3^ Key Laboratory of Terahertz Solid State Technology Shanghai Institute of Microsystem and Information Technology Chinese Academy of Sciences Shanghai 200050 China; ^4^ Department of Mechanical Engineering the University of Texas at Austin Austin TX 78712 USA; ^5^ Department of Neurosurgery Huashan Hospital of Fudan University Wulumuqi Zhong Road 12 Shanghai 200040 China; ^6^ School of Physical Science and Technology ShanghaiTech University Shanghai 200031 China; ^7^ Department of Physics and Astronomy Stony Brook University Stony Brook NY 11794 USA; ^8^ Department of Biomedical Engineering Tufts University Medford MA 02155 USA

**Keywords:** metamaterials, multicolor imaging, T‐ray imaging

## Abstract

Recent progress in ultrafast spectroscopy and semiconductor technology is enabling unique applications in screening, detection, and diagnostics in the Terahertz (T‐ray) regime. The promise of efficaciously operation in this spectral region is tempered by the lack of devices that can spectrally analyze samples at sufficient temporal and spatial resolution. Real‐time, multispectral T‐ray (Mul‐T) imaging is reported by designing and demonstrating hyperspectral metamaterial focal plane array (MM‐FPA) interfaces allowing multiband (and individually tunable) responses without compromising on the pixel size. These MM‐FPAs are fully compatible with existing microfabrication technologies and have low noise when operating in the ambient environment. When tested with a set of frequency switchable quantum cascade lasers (QCLs) for multicolor illumination, both MM‐FPAs and QCLs can be tuned to operate at multiple discrete THz frequencies to match analyte “fingerprints.” Versatile imaging capabilities are presented, including unambiguous identification of concealed substances with intrinsic and/or human‐engineered THz characteristics as well as effective diagnosis of cancerous tissues without notable spectral signatures in the THz range, underscoring the utility of applying multispectral approaches in this compelling wavelength range for sensing/identification and medical imaging.

Terahertz spectroscopy and imaging techniques have been applied to many areas such as molecular spectroscopy,^1^, ^2^, ^3^ security imaging,^4^, ^5^, ^6^ and medical diagnosis.^7^, ^8^, ^9^ A wide range of chemical and biological agents, such as explosives, drugs, and tissues have unique spectral signatures at THz frequencies,^4^, ^7^, ^10^, ^11^ which can be unambiguously identified using THz spectroscopy. THz radiation can penetrate deeply into nonpolar and nonmetallic materials that are usually opaque at optical wavelengths, permitting examination of concealed hazardous substances. Moreover, great opportunities for T‐ray imaging technologies can be found in medical diagnostics (e.g., cancerous tissues) because THz radiation is nonionizing and therefore inherently safe for the human body.^12^ Moreover, THz photons are also highly sensitive to water molecules so small variations in hydration levels could be a critical measure in determining normalcy for biological systems.

In general, THz imaging uses two detection methods: coherent detection and incoherent detection. Coherent detection typically measures the electric‐field of the broadband THz pulses in time domain using nonlinear crystals or photoconductive antennas;^13^ while incoherent detection measures the time‐integrated THz power utilizing sensors such as bolometers,^14^, ^15^ Golay cells,^16^ Schottky diode‐based detectors,^17^ field‐effect transistor (FET)‐based detectors,^18^ and THz quantum well photodetectors.^19^ While both detection methods can find successful applications, fast real‐time THz imaging with fine spectral and spatial resolution is considerably more challenging. For example, imaging with pulsed THz‐time domain spectroscopy (TDS) yields broadband spectrum but is hard to scale up to large array detection. In addition, THz TDS requires a complete scan in time domain to yield the essential Fourier transformed spectrum, which can be time consuming because a finer spectral resolution requires a longer scan. Incoherent detection, on the other hand, often lacks the frequency tunability and is bulky in size, therefore shrinking both the dimensions of the THz source and the detector can be quite challenging. An ideal THz imager should inherit the merits of both detection methods and simultaneously offer a fast response to the desired frequency bands, permit large area detection in a compact footprint, and work preferably in the ambient environment without cryogenic cooling. Innovations in materials and designs along these directions have yet to be explored extensively.

Natural materials do not show much response in the THz frequency range. Metamaterials (MMs)—artificial structures in which the electromagnetic responses can be engineered by design to access a frequency range normally inaccessible to natural materials—can show strong responses (for example, high absorption) in the THz regime and have the potential of “filling the THz gap.”^20^, ^21^ MM‐based focal plane arrays (MM‐FPAs) can provide fast and efficient T‐ray imaging with great spectral selectivity, high power sensitivity, narrowband responsivity, and good linearity at room temperature, allowing a flexible and inexpensive route to perform imaging using designer sensor arrays based on MMs.^22^, ^23^, ^24^, ^25^, ^26^, ^27^ Here, we report a real‐time multispectral T‐ray (Mul‐T) imager using a multispectral MM‐FPA, which can be readily tuned to selectively detect the incident T‐rays at discrete frequency bands for multicolor imaging. By integrating distinct single‐band metamaterial unit cells together, multiple bands can be readily achieved, which is geometrically scalable to cover multiple frequencies on demand.^28^ All of the resonances of the multispectral MMs can be tuned independently. We note that acquiring a useful THz image for the purpose of sample identification may not require the full spectral information—only contrasts (in the transmission, reflection and/or absorption spectrum) at several “fingerprint” frequencies are necessary. By coinciding the resonant responses of MMs with those of targeted substances (such as explosives, hazardous chemicals and biological compounds), a multicolor THz imager allows the target to be identified more accurately because there are multiple frequency‐match points of comparison.^29^

In this work, we use this Mul‐T imaging technology to non‐destructively identify pesticide residues and medication substances under concealment where visual inspection is not possible. A set of home‐built frequency‐switchable THz quantum cascade lasers (QCLs)^30^, ^31^ are integrated to allow active THz illumination with fast frequency switching and fine spectral width tuning. The operating frequencies—including both the multispectral MM‐FPA and the THz QCL sources—can be readily tuned to match the “fingerprints” of analytes for fast screening. For example, menadione (a nutritional supplement with Vitamin K activity), copper oxalate (a major ingredient of some pesticide), and benzylpenicillin potassium (a type of antibiotics), with spectral signatures at 2.5 THz, 3.4 THz, and 4.3 THz, respectively, are chosen as proof‐of‐principle demonstrations. In addition, we demonstrate that cancerous brain tissues can be successfully resolved from normal tissues, with both of which showing no clear THz signatures, but a different spectral integrated THz contrast due to synergistic effects of variations in the composition, density, and hydration level.

The Mul‐T imaging setup using a THz multispectral MM‐FPA and a set of QCL sources is schematically illustrated in **Figure** **1**A. A group of QCL sources, emitting at different frequencies, each with a lasing frequency bandwidth of ≈150–300 GHz (Figure S1, Supporting Information), are developed in‐house and can essentially cover a wide frequency range from 2.5 to 5 THz by integrating more QCL chips. The MM‐FPAs (using multispectral metamaterial array as the sensing element), which absorb THz radiation at multiple bands, can then be paired with tunable QCL sources in accordance with different applications. This novel combination with good flexibility offers a platform that is capable of producing Mul‐T images of the targets by superimposing multiple monolithic (binary or grayscale) images acquired from a single MM‐FPA at the selected frequencies. The targets can then be identified via correlating the THz images to their THz signatures.

**Figure 1:**
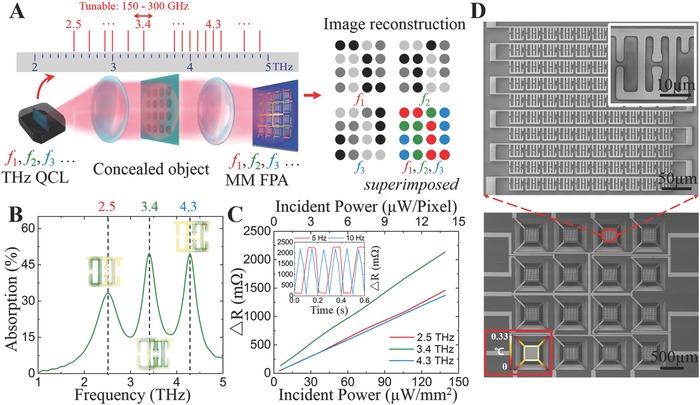
Multispectral T‐ray (Mul‐T) imaging using multispectral metamaterial focal plane arrays (MM‐FPAs). A) Schematic description of Mul‐T imaging. A set of QCL sources emitting at different dominant frequencies with typically 150–300 GHz tuning range essentially covers the continuous spectra in THz frequencies from 2.5 THz to 5 THz. The frequency “ruler” shows available home‐built THz QCL sources. B) Numerical simulation of the absorption of the MM‐based multispectral THz detector, three distinct absorption peaks are found at 2.5, 3.4, and 4.3 THz, respectively. (Inset) Simulated surface current density distributions when on‐resonance. All three LC resonances originate from circulating currents in individual single‐band resonators. C) The response of individual pixels characterized as a function of incident power irradiated by THz QCL sources. The responsivity with 2.5, 3.4, and 4.3 THz illuminations are measured to be 1.05 × 10^5^, 1.56 × 10^5^, and 1.00 × 10^5^ Ω W^−1^, respectively. (Inset) The temporal responses characterized by 3.4 THz QCL source modulated at frequencies of 5 Hz (red) and 10 Hz (blue), respectively. D) SEM images of a portion of the Mul‐T MM‐FPA detector. (Inset) Thermal simulation of an individual pixel upon irradiation with the assumption of 100 µW mm^−2^ incident power and 30% photothermal conversion efficiency. An increase by ≈0.33 °C of temperature is calculated.

For Mul‐T imaging, we choose three operating frequency bands centered at 2.5, 3.4, and 4.3 THz, respectively, which are in accordance to the THz spectral signatures of menadione, copper oxalate, and benzylpenicillin potassium as proof‐of‐principle demonstrations. The triple‐band MM is designed to selectively absorb at these three frequency bands using a commercial finite‐difference time domain solver CST Microwave Studio (Figure 1B) (see Figure S2 in the Supporting Information for more details). Note that more absorption bands can be readily added by following the same design paradigm (Figure S3, Supporting Information).^32^, ^33^, ^34^ In fact, the addition of absorption bands does not considerably compromise the resolution of the detector, a unique feature enabled by as‐reported multispectral MM‐FPAs compared to other technologies (e.g., stitching multiple resonator arrays^35^ or stacking multiple resonators^36^) for multispectral THz imaging. The simulated surface current density distributions (Inset of Figure 1B) at individual resonant frequencies (LC resonances) reveal that the circulating currents of each single band resonator are mostly decoupled from adjacent ones when on resonance, allowing fine tuning of each of the frequency‐band response (Figure S4, Supporting Information).

The device performance is experimentally evaluated in terms of the responsivity (Figure 1C) and transient response (Inset of Figure 1C). The responsivity, obtained by plotting the resistance of the device against the incident power, is calculated to be 1.05 × 10^5^, 1.56 × 10^5^, and 1 × 10^5^ Ω W^−1^ at 2.5, 3.4, and 4.3 THz, respectively. The output noise voltage spectral density is measured utilizing a low‐noise current preamplifier (Stanford Research, SR570, USA) and a spectrum analyzer (Agilent, N9020A, USA). The noise equivalent power (NEP), defined as the ratio between the output noise voltage and detector voltage responsivity, is calculated to be 133, 86, and 134 pW Hz^−1/2^ at 2.5, 3.4, and 4.3 THz, respectively. The curves show reasonable linearity within the range of tested incident power thanks to the linear temperature coefficient of resistance (TCR) of platinum (Pt) (Figure S5, Supporting Information). The device shows the best performance at 3.4 THz in terms of responsivity, which is in reasonable agreement with the transmission/absorption spectra (Figure S6, Supporting Information). Meanwhile, the transient response of the 3.4 THz QCL, modulated at 5 Hz (red) and 10 Hz (blue), shows a maximum operation frequency at 10 Hz before the signal rolls off. The transient responses of the device operating at 2.5 and 3.4 THz show similar results (Figure S7, Supporting Information). The short response time offers remarkable potential for applications that require real time measurements. As a thermal detector, faster responses (e.g., 30 fps for most real‐time imaging) can be achieved at the expense of the responsivity (Figure S8, Supporting Information).^37^, ^38^ The high responsivity, low NEP, and fast response time of the MM‐FPA at all three frequencies justify this device as a favorable alternative to other THz thermal detectors with similar NEP levels (≈100 pW Hz^−1/2^, 2–5 THz) such as Golay cells (which are difficult to scale up) and far‐infrared cameras (which lack spectral selectivity) for real‐time Mul‐T imaging applications (Table S1, Supporting Information).

The THz MM‐FPAs are fabricated using the standard microfabrication process with a front‐side‐release bulk‐silicon technique (Figure S9, Supporting Information). The pixels of the MM‐FPA are fabricated on thermally insulating silicon nitride cantilevers. They are mechanically independent of each other and can be easily scaled up. A prototype of MM‐FPA consisting of 4 × 4 pixels is fabricated, where each pixel contains an electrically interconnected MM array consisting of 10 × 10 unit cells, which is optimized for detection sensitivity and speed in the current set up (Figure 1D). The absorption of THz wave generates heat in the Pt MMs and raises the temperature of the interconnected MM array, which in turn alters the electrical resistance due to the thermal resistance effect. The variation in the resistance is then measured by the readout module and correlated back to obtain the incident THz radiation. In general, there is a trade‐off between the response speed and sensitivity of a thermal detector. Thermal isolation allows longer integration time to detect weaker signals; however, the detector response time is unavoidably increased. The thermal simulation of a pixel of the MM‐FPA in the ambient environment is demonstrated in the inset of Figure 1D by assuming 100 µW mm^−2^ incident power and 30% photothermal conversion efficiency. An increase by 1660 mΩ of the resistance—which corresponds to 0.33 °C increase of temperature—and a thermal equilibrium time of ≈100 ms are obtained from simulation results, which is close to the measured resistance change of 1590 mΩ at 3.4 THz (extracted from the measured responsivity curve in Figure 1C) with the same incident power.

The development of reliable and economically viable T‐ray imaging systems is largely challenged by the lack of available sources (for active imaging) and appropriate sensing elements (for both active and passive imaging). For example, commercially available products such as FET‐based THz cameras (mostly working at frequencies <1 THz) and mid‐IR camera (most optimized for operating at the mid‐IR window between 7.5 and 14 µm) have their limitations.^39^, ^40^, ^41^ The proposed MM‐based FPA detector can be readily tuned to cover the entire THz regime. To investigate the imaging capability of the fabricated MM‐FPAs, three set of experiments are carried out, including 1) direct imaging of a focused THz beam profile; 2) binary imaging of metallic objects (highly reflective at THz frequencies); 3) grayscale imaging of MMs with human‐engineered electromagnetic responses at multiple THz frequencies.

As shown in **Figure** **2**A, the MM‐FPA successfully captures the intensity profile of a focused QCL THz beam operating at 3.4 THz. A Gaussian intensity distribution is observed which is consistent with the result acquired by a commercial THz camera (see Figure S10, Supporting Information, NEC IRV‐T0831C, Japan; Note that an IR blocking filter is necessary to reduce the noise in the NEC camera due to its broadband IR response/absorption), demonstrating the utility of the MM‐FPA as a reliable and simple‐to‐use tool for THz beam quality evaluation and diagnosis. A preliminary demonstration of metal detection is conducted by real‐time binary imaging of “T,” “H,” and “Z” letters patterned in gold on a 400 nm thick silicon nitride thin film. The captured binary images @ 3.4 THz—similar results are obtained at 2.5 and 4.3 THz—clearly reveal the metallic patterns (Figure 2B). Additionally, the spectrally selective imaging capability of the MM‐FPA detector can be applied to the detection of distinctive line structures of many molecular species, which are mimicked by using a set of patterns composed of artificially designed MM structures with engineered characteristic transmission spectra at THz frequencies. The artificial structures consist of a series of split ring resonators (SRRs) and complementary SRRs (CSRRs) whose “fingerprints” in the THz regime can be finely tuned via optimization of their geometries. For consistency, 2.5, 3.4, and 4.3 are selected as operation frequencies for proof of concept demonstrations. As shown in Figure 2C, the top row of “T,” “H,” and “Z” patterns consist of the SRR structures (as opposed to solid patterns in the binary imaging experiments) with resonances (i.e., the minimum transmission) centered at 2.5, 3.4, and 4.3 THz while the bottom row consists of their respective CSRR structures at the same resonant frequencies (i.e., the maximum transmission). The multispectral grayscale imaging of the six MM patterns is then carried out using the single MM‐FPA with the electric field perpendicular to the SRR gap and parallel to the CSRR gap (the electric field is rotated by 90°).^42^ The intensity values of the “T,” “H,” and “Z” letters on the images taken at the three THz frequencies correlate well with the measured transmission spectra. For example, at 3.4 THz, the top row “T” and “Z” letters show similar signal intensity due to the similar measured transmission. Meanwhile, the “H” letter shows less intensity since the transmission of the metamaterial in that area is lower. On the bottom row, the signal intensity of “H” is higher than “T” and “Z,” as verified by the higher transmission of the corresponding CSRR metamaterial. The multispectral images at the other two frequencies are also consistent with the measured spectra of artificially engineered MM patterns (Figure 2D).

**Figure 2:**
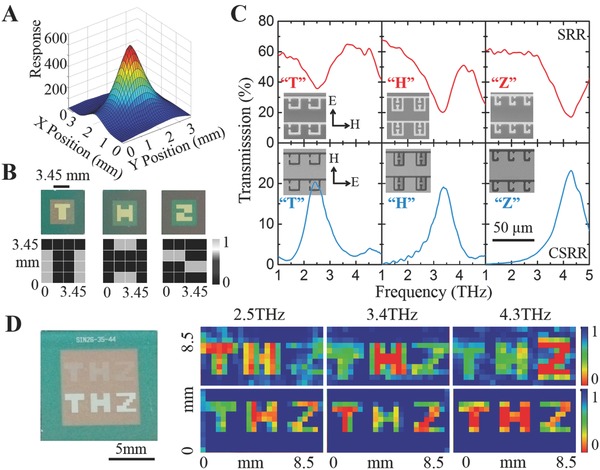
Binary and grayscale T‐ray imaging. A) Real‐time imaging of the incident THz beam profile of a 3.4 THz QCL source. The beam profile is consistent with the result acquired by a commercial THz camera. B) Real‐time binary T‐ray imaging (bottom row) of a mask (top row) with “T,” “H,” and “Z” letter shapes (top row) patterned in gold on a 400 nm thick silicon nitride thin film at 3.4 THz. C) Measured transmission spectra of MM arrays. (Inset) SEM images of a portion of SRR‐based and CSRR‐based MMs consisting the “THZ” patterns. D) Multispectral grayscale T‐ray imaging of “THZ” patterns consisting of MM arrays with human‐engineered THz characteristics. The color scale is normalized to a maximum transmission of 1.

Mul‐T imaging can be a powerful tool for nondestructive and noncontacting testing to inspect sealed packages for identification of concealed materials by being able to not only “see through” the package to locate the target but also to identify what it is. In this work, nondestructive spectroscopic identification of various drug tablets concealed by 5 mm thick polytetrafluoroethylene (PTFE, also known as Teflon) envelope is performed using a Mul‐T MM‐FPA. **Figure** **3**A shows the tablets used for Mul‐T imaging where PTFE powders are employed as an excipient. The significant ingredients of the tablets are menadione (a nutritional supplement with Vitamin K activity), copper oxalate (a major ingredient of some pesticide) and benzylpenicillin potassium (a type of antibiotics), which show distinct spectral signatures at 2.5 THz, 3.4 THz and 4.3 THz (characterized using THz time‐domain spectroscopy (THz‐TDS)), respectively (Figure 3B). The tablets are concealed in a PTFE envelope and are visually inaccessible (Figure S11, Supporting Information). However, their profiles clearly appear in all three THz images (grayscale images) captured at their respective characteristic frequencies using the Mul‐T MM‐FPA. To visually reconstruct the spectral information conveyed from the THz images, the three grayscale images are processed in false‐colors (2.5 THz, red; 3.4 THz, green; 4.3 THz, blue) and then superimposed to form a full 24 bit RGB color image, from which the specific ingredients of the concealed samples can be easily distinguished (Figure 3C). The intentionally created crack (of ≈100 µm) in the tablet I (menadione) and the boundary line between two materials in the tablet IV (menadione and copper oxalate) can be clearly resolved, underscoring the good spatial resolution of our MM‐FPA device. However, limited by the diffraction, T‐ray imaging system cannot provide a better spatial resolution compared with the imaging systems working at higher frequency, such as infrared or visible imaging system.

**Figure 3:**
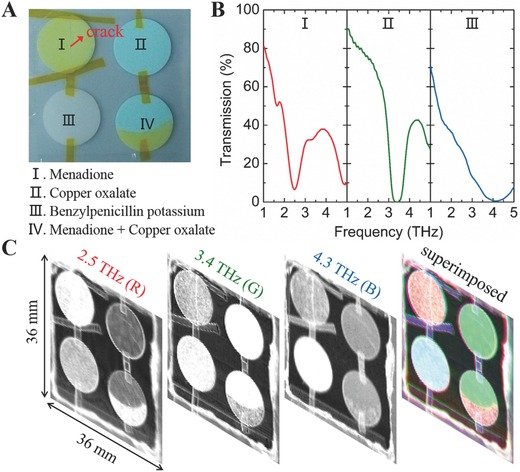
Mul‐T imaging for non‐destructive material identification and defect inspection. A) Tablets used for Mul‐T imaging. The significant ingredients are menadione (I, a nutritional supplement with Vitamine K activity), copper oxalate (II, a major ingredient of some pesticides), and benzylpenicillin potassium (III, a kind of antibiotics), respectively. There is an intentionally created crack in tablet I. Tablet IV contains two kinds of material (I and II) with a clear boundary line. B) Transmission spectra of the tablets with unique “fingerprints” at THz frequencies. C) Mul‐T imaging for the tablets concealed by PTFE at 2.5 THz (Red), 3.4 THz (Green) and 4.3 THz (Blue), from which the specific ingredients of the concealed samples can be easily distinguished. The crack and the boundary line in tablet I and tablet IV are clearly reproduced in the full color image.

The previously established and widely used techniques for the advanced medical imaging are magnetic resonance imaging (MRI) and computed tomography (CT). However, MRI tests usually take a long time (≈20–45 min) for a typical brain scan and CT uses ionizing radiation, which is considered to be invasive to the human tissues. Differently, the technique presented in this work (i.e., the multispectral MM‐based THz imaging system) uses nonionizing THz radiation to ensure the biological safety. Note that interpretation of medical images often poses daunting challenges unless the relevant spectral signatures of different tissue types are available. However, the capability of operating at multiple frequencies using Mul‐T MM‐FPAs (which can be readily designed and easily implemented) provides a facile way for medical diagnosis (and complementary to other, conventional imaging techniques) as long as there is enough contrast (at certain THz frequencies) due to synergistic effects of the elemental composition, density, hydration level, and protein conformation of targeted biological substances, even if there are no significant spectral characteristics in these individual substances (e.g., normal and cancerous tissues) in the THz regime.

For a proof‐of‐principle demonstration, contact‐less identification of metastatic brain tumors from the normal tissues is carried out using the multispectral MM‐FPA‐based THz imaging system. Metastatic brain tumors are among the most common brain tumors in humans and patients rarely survive more than 6 months.^43^ **Figure** **4**A shows the sagittal T1‐weighted MRI image of a metastatic brain tumor from a 57 year old female patient with a medical history of lung neoplasm resection. The tumor sample is collected in the area where tumor cells conglomerate next to the normal brain tissue. The metastatic brain tumor is visually indistinguishable from the normal tissue, unless being stained with hematoxylin and eosin (H&E) (Figure S12, Supporting Information). As shown in Figure 4B, there are no significant spectral characteristics in the metastatic brain tumors and normal brain tissues in the THz regime, however, a substantial spectral contrast exists in the differential THz spectra (obtained from subtracting the transmission intensity of normal tissue by the cancerous one). A set of THz images of the brain tissue containing metastatic tumors is then captured at 2.5, 3.4, 4.0, and 4.3 THz, respectively. Note that the image at 4.0 THz is captured by an additional single spectral THz detector optimized at 4.0 THz and a 4.0 THz QCL (Figure S13, Supporting Information). As shown in Figure 4C, the highest contrast is observed at 4.3 THz, which is consistent with their THz spectra measured using THz‐TDS in Figure 4B. On the other hand, although the THz imaging technique for in vivo applications has been hindered by the limited penetration depth of THz wave due to the strong absorption of water, the prospective of more powerful THz sources could greatly alleviate this issue and make the THz imaging a suitable technique in more clinical situations.

**Figure 4:**
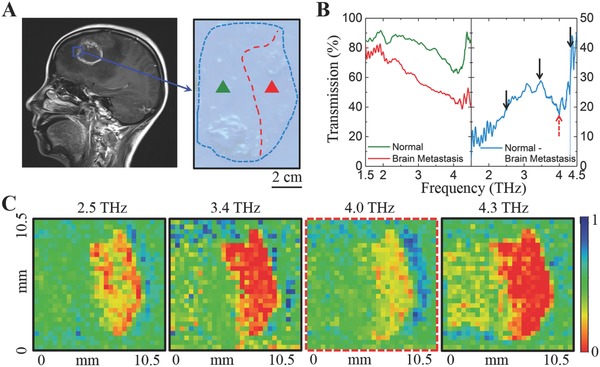
Mul‐T imaging for effective diagnosis of cancerous brain tissues. A) Sagittal T1 weighted MRI image of a patient with a metastatic brain tumor. The tumor sample is collected containing the boundary between the metastatic brain tumor and normal brain tissue. The metastatic brain tumor is optically transparent and visually indistinguishable from the normal brain tissue. B) Transmission spectra of the normal tissue (green line) and brain metastasis (red line). The blue line shows the transmission difference between the transmission of normal tissue and brain metastasis. C) Multi‐T imaging for the tumor sample. The THz images of the sample are collected at frequencies of 2.5, 3.4, 4.0, and 4.3 THz. The image at 4.0 THz is captured by an additional single spectral THz detector optimized at 4.0 THz. The highest contrast between normal tissue and brain metastasis is observed at 4.3 THz, which is consistent with the measured THz spectra. The color scale is normalized to a maximum transmission of 1.

Most MM‐based THz detectors or imagers (including the device reported in this work) are inherently thermal detectors whose performances largely depend on the efficiency of converting radiation energy to heat and then to an electrical readout signal. It is difficult to find strongly absorbing materials at THz frequencies that are compatible with standard microfabrication techniques so to be scaled up easily for large array imagers. Several materials, such as VOx,^44^ poly‐Si‐Ge,^45^ and YBCO,^46^ have been utilized within the sensing element in many commercially available thermal detectors, some of which have been used for THz detection/imaging. Their responses are typically optimized for mid‐IR (≈7.5–14 µm) operation, with considerably weaker responses at THz frequencies (≈30–3000 µm).^40^, ^47^ Additionally, most of these materials show a broadband response. This limits potential applications, such as spectrally selective detection of explosive materials, which show unique responses at varied THz frequencies.

MM unit cells are typically λ/5–λ/10 in size and fabricated in array form, making them suitable for real‐time 2D imaging. It is straightforward to combine multiple MM structures (3 in this work, however more are feasible) with desired resonant frequencies into a “super‐pixel” (while still within the diffraction limits of λ/2). However, one single multispectral MM‐FPA allows multi‐band responses (and individually tunable) without compromising on the pixel size (*∧* ≅λ/4, as reported in this work)—no need for stitching multiple resonator arrays or stacking multiple resonators. Moreover, tunable multispectral hybrid metamaterials that enable optically or electrically modulated “frequency agile multicolor” imaging is also possible by combining the multispectral metamaterial structures with semiconducting materials or phase change materials.^48^, ^49^, ^50^ It is worth mentioning that the Mul‐T MM‐FPA imager can operate—and is characterized in this work—at room temperature and ambient pressure. Cryogens are only used for cooling down the QCL sources. The power sensitivity is comparable, if not favorable, to existing commercial thermal detectors for broadband THz sensing and could be further improved by design optimization and additional engineering efforts.^51^, ^52^ An additional layer can also be introduced (in the fashion of double layer “perfect absorbers”) to enhance the THz power absorption at the expense of moderately increasing the thermal mass.^53^

In summary, we have demonstrated real‐time multispectral T‐ray imaging using QCLs as multicolor illumination sources and multispectral MM‐FPAs as MEMS‐compatible and CMOS‐integrable detection elements.^54^, ^55^ Both QCLs and MM‐FPAs can be readily tuned to operate at multiple discrete THz frequency bands to match the characteristic frequency signatures of the sample. We foresee that the combination of conventional THz‐TDS (for collecting the THz spectral features of target substances of interest and consequently building the “THz fingerprint library”) and the THz MM‐FPAs (for direct imaging at these spectral signatures) would provide a safer and more efficient technique for security screening and medical diagnosis.

## Experimental Section

*THz MM‐FPA Detector Design*: The electromagnetic simulations were carried out using the finite‐integration time domain solver of the CST Microwave Studio. The program simulated a single unit cell as shown in Figure 1B. Perfect magnetic (PM) and perfect electric (PE) boundary conditions were applied along the *x*‐ and *y*‐directions (the gap was along the *y*‐direction). Waveguide ports on the other boundaries simulated a TEM plane wave propagating through the medium. The THz wave was normally incident on the metamaterial. The thermal simulations were carried out using the heat transfer module of the commercial finite element software COMSOL Multiphysics. Temperature boundary conditions are applied on the interface between the supporting membrane and the bulk silicon, and heat flux boundary conditions are applied on the remaining boundaries.

*THz MM‐FPA Detector Fabrication*: A low‐stress silicon nitride thin film with a thickness of 800 nm was first deposited on a (100) silicon substrate. The Cr/Pt layer (10/100 nm) defining the SRR array was then fabricated using a standard UV lithography followed by an ion beam etching process. Next, 30/300 nm Cr/Au defining the wire was performed using a standard liftoff process. Subsequently, the silicon nitride layer was patterned using the UV lithography and plasma etching processes to define the SRR array supporting membrane. Finally, the structures were released through TMAH wet etching of the silicon underneath the SRR array from the front side.

*THz QCL Fabrication*: The structures of the THz QCLs used in this work were grown by the molecular beam epitaxy (MBE). The growth starts with a 400 nm thick n‐type GaAs bottom contact layer, which is silicon‐doped to 3 × 10^18^ cm^−3^. Then, the active region core structure was grown on top of the bottom contact layer. The THz QCL active region structures for emitting at 2.5, 3.4, and 4.3 THz are different. For 2.5 THz lasing, the active region is based on the bound‐to‐continuum transitions,^56^ while for 3.4 and 4.3 THz emission, the QCL active region is based on resonant phonon design.^57^ On top of the active region, a 50 nm thick n‐type GaAs top contact layer with a doping of 5 × 10^18^ cm^−3^ was grown. After the growth, the MBE‐grown wafers were processed into single‐plasmon waveguide ridge lasers. First, the optical lithography and inductively coupled plasma etching were used to define and etch the ridge structures. The top (Ti/Au, 10/300 nm) and lateral (Ge/Au/Ni/Au, 13/33/30/300 nm) metallic electrodes were fabricated employing the electron‐beam evaporation and lift‐off techniques. In order to form the ohmic contacts, the lateral metal was annealed at 370 °C for 40 s. Then, the semi‐insulating substrate was thinned down to 150 µm to improve the temperature performance of the devices. Finally, the fabricated ridge structures were cleaved to small laser bars with the cavity length ranging from 1 to 4 mm. The as‐cleaved laser bar was indium‐soldered on a copper heat sink. The electrical injection was achieved using wire bonding to connect the laser chips to the contact pads.

*MM‐Based “THZ” Pattern Fabrication*: A low‐stress silicon nitride thin film with a thickness of 800 nm was first deposited on a (100) silicon substrate. Then etching windows on the back surface silicon nitride layer was defined using the UV lithography and plasma etching processes. Next, the Cr/Pt layer (10/100 nm) defining the SRR array was fabricated using a standard UV lithography followed by an ion beam etching process. Finally, the structures were released through TMAH wet etching of the silicon underneath the SRR array from the back side.

*Tablets Preparation*: For sample preparation, menadione, copper oxalate, and benzylpenicillin potassium were purchased with 99% purity. These samples were formed into tablets with PTFE powder at the concentration of 5 wt%. Samples and PTFE powder were mixed and pressed to reach the final thickness of 0.6 mm and diameter of 13 mm.

*Hematoxylin and Eosin (H&E) Staining*: The paraffin sections were first deparaffinized and rehydrated. Then they were washed briefly in deionized water. Next, they were stained in Harris hematoxylin solution for 8 min and washed in running tap water for 5 min. Subsequently, they were differentiated in 1% acid alcohol for 30 s and washed in running tap water for 1 min twice. Then the hematoxylin stained tissues were counterstained in eosin–phloxine solution for 30 s and dehydrated through 95% alcohol for 5 min three times. The stained tissues were cleared in xylene for 15 min three times and mounted with xylene based mounting medium. Finally, they were dried overnight in a fume hood.

*THz Spectra Measurement*: The THz spectra were measured using a commercial asynchronous‐optical‐sampling THz‐TDS system TAS7500TS (Advantest, Japan) with a resolution of 7.6 GHz, spectral range from 1 to 5 THz. It is continuously purged with dry air. In the measurement, MM samples were mounted at a normal incident angle with the electric field perpendicular to the gap of the metamaterial. And the free space measurement is defined as the reference.

*CW Spectra of THz QCLs Measurement*: The CW spectra of THz QCLs were measured using a Fourier transform infrared (FTIR) spectroscopy V80 (Bruker, Germany) with a spectral resolution of 3 GHz. A THz beam splitter (Mylar) and a far‐infrared deuterated triglyine sulfate (DTGS) detector are used. The optical chamber of the FTIR is pumped down to 2 mbar.

*THz MM‐FPA Detector Characterization*: To calibrate the sensitivity of the THz MM‐FPA detector, the power of the THz QCL with different modulation frequencies were first measured. Then the average power of the THz QCL was measured by a THz power meter (Ophir 3A‐P THz) using a standard power measurement geometry for collecting and collimating the THz light onto the detection element. Once the THz light power was measured, the THz power meter was replaced by the THz MM‐FPA detector for various characterizations. Here, the polarized THz wave emitted from the THz QCL was normally incident on the MM‐FPA detector with the electric field perpendicular to the gaps of the metamaterials. It is worth noting that to simultaneously satisfy the requirements for the pulse operation of THz QCLs and slow response of the THz MM‐FPA detector, a double‐modulation technique was employed in this measurement. First of all, to guarantee the strong THz power emission from THz QCLs, a fast electrical modulation was applied on THz QCLs using a pulse generator with a repetition rate of 10 kHz and a duty cycle ranging from 1% to 40%. However, the 10 kHz modulated THz light cannot be detected by the THz FPA detector. Therefore, a second slow modulation was applied on THz QCLs, which was achieved by setting the pulse generator in “Gated” mode and sending the slow modulation signal with a frequency between 1 and 10 Hz from a wave function generator to the pulse generator.

## Conflict of Interest

The authors declare no conflict of interest.

## Supporting information

Supplementary
