# Supplementary material for: Multicolor T‐Ray Imaging Using Multispectral Metamaterials
Source: Adv Sci (Weinh). 2018 Mar 25;5(7):1700982. doi: 10.1002/advs.201700982 (PMC6051390; doi:10.1002/advs.201700982)
Supplement: Supplementary file 1 — Supplementary [file ADVS-5-1700982-s001.pdf]

Copyright WILEY-VCH Verlag GmbH & Co. KGaA, 69469 Weinheim, Germany, 2018.

ADVANCED SCIENCE
Open Access

Supporting Information

for *Adv. Sci.,* DOI: 10.1002/advs.201700982

Multicolor T-Ray Imaging Using Multispectral Metamaterials

Zhitao Zhou, Tao Zhou, Shaoqing Zhang, Zhifeng Shi, Ying Chen, Wenjian Wan, Xinxin Li, Xinzhong Chen, Stephanie N. Gilbert Corder, Zhanglong Fu, Liang Chen, Ying Mao, Juncheng Cao, Fiorenzo G. Omenetto, Mengkun Liu, Hua Li,* and Tiger H. Tao**

Copyright WILEY-VCH Verlag GmbH & Co. KGaA, 69469 Weinheim, Germany, 2013.

Supporting Information

Multicolor T-ray imaging using multispectral metamaterials

Zhitao Zhou, Tao Zhou, Shaoqing Zhang, Zhifeng Shi, Ying Chen, Wenjian Wan, Xinxin Li, Xinzhong Chen, Stephanie N. Gilbert Corder, Zhanglong Fu, Liang Chen, Ying Mao, Juncheng Cao, Fiorenzo G. Omenetto, Mengkun Liu, Hua Li*, Tiger H. Tao**

Zhitao Zhou, Ying Chen, Prof. Xinxin Li, Prof. Tiger H. Tao
State Key Laboratory of Transducer Technology, Shanghai Institute of Microsystem and Information Technology, Chinese Academy of Sciences, Shanghai, 200050, China

Zhitao Zhou, Prof. Xinxin Li, Prof. Juncheng Cao, Prof. Hua Li, Prof. Tiger H. Tao
School of Graduate Study, University of Chinese Academy of Sciences, Beijing 100049, China

Tao Zhou, Wenjian Wan, Zhanglong Fu, Prof. Juncheng Cao, Prof. Hua Li
Key Laboratory of Terahertz Solid State Technology, Shanghai Institute of Microsystem and Information Technology, Chinese Academy of Sciences, Shanghai, China.
Email: jccao@mail.sim.ac.cn; Email: hua.li@mail.sim.ac.cn

Zhifeng Shi, Prof. Liang Chen, Prof. Ying Mao
Department of Neurosurgery, Huashan Hospital of Fudan University, Wulumuqi Zhong Road 12, Shanghai, 200040, China

Xinzhong Chen, Stephanie Gilbert Corder, Prof. Mengkun Liu
Department of Physics and Astronomy, Stony Brook University, Stony Brook, New York 11794, USA

Prof. Xinxin Li, Prof. Tiger H. Tao
School of Physical Science and Technology, ShanghaiTech University, Shanghai 200031, China

Prof. Fiorenzo G. Omenetto
Department of Biomedical Engineering, Tufts University, Medford, MA 02155, USA

Shaoqing Zhang, Prof. Tiger H. Tao
Department of Mechanical Engineering, the University of Texas at Austin, Austin, TX 78712, USA
E-mail: tiger.tao@austin.utexas.edu

Keywords: T-ray imaging, metamaterial, multicolor

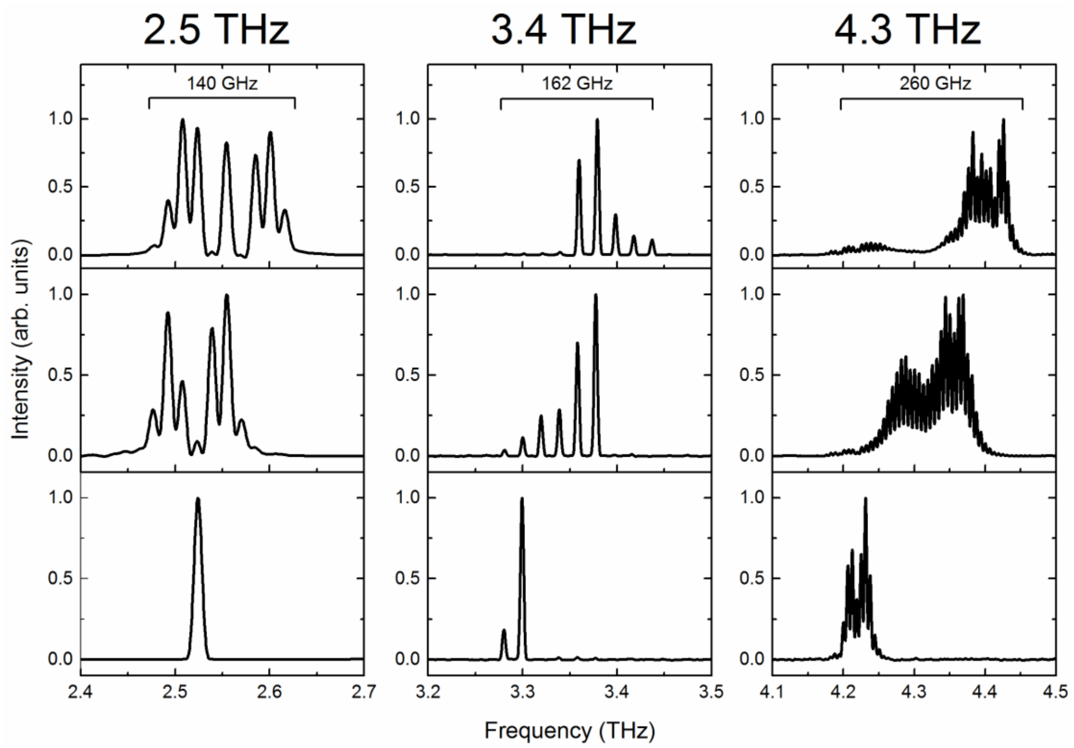

Figure S1. The lasing frequency bandwidths of the three THz QCL sources. The left, middle, and right column show the emission spectra of THz QCLs emitting around 2.5, 3.4, and 4.3 THz, respectively. For each column, from bottom to up the drive current increases from threshold to maximum current. The measured frequency coverage in the whole current dynamic range is defined as lasing bandwidth which is measured to be 140, 162, 260 GHz for the 2.5 THz, 3.4 THz, and 4.3 THz QCL, respectively. The THz emission spectra were measured using a Fourier transform infrared spectrometer under vacuum.

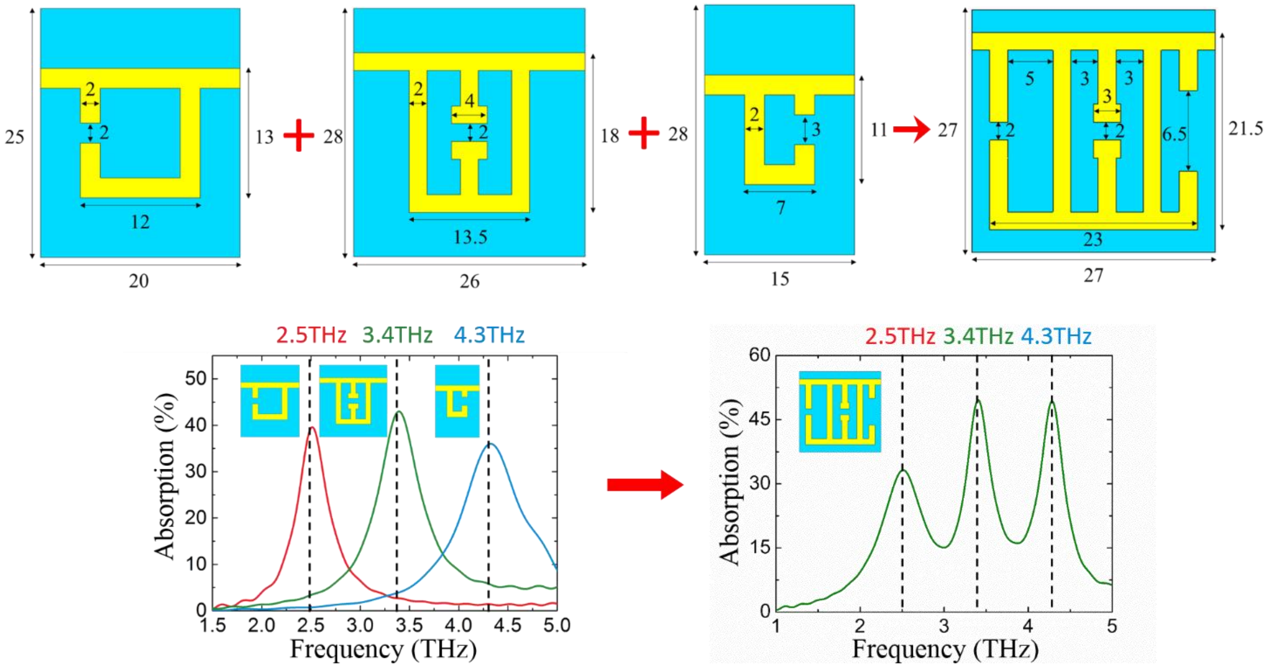

Figure S2. The design concept of hyperspetral metamaterial. The fundamental structure of THz metamaterial is a metallic ring with a gap etched on a dielectric substrate, which can provide continuously tunable and controllable inductive-capacitive (LC) resonance. To obtain multi-band THz metamaterial, we can integrate several single-band metamaterial unit cells together. And simulation results demonstrate that the size of the integrated multi-band THz metamaterial unit cell is as similar as those single-band ones. Such an advantageous fact indicates that we can achieve large multi-spectral THz FPA detector at the expense of moderately increasing the size of the detector. All units are in microns.

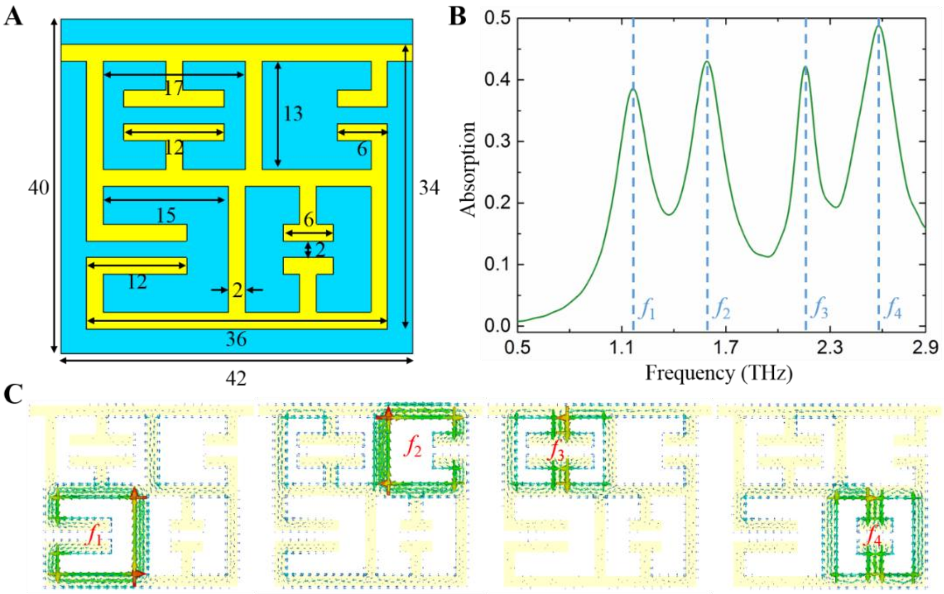

Figure S3. The design of quadruple-band THz metamaterial. (A) Dimensions of the designed four-band THz metamaterial. All units are in microns. (B) Numerical simulation of the absorption of the quadruple-band THz metamaterial, four distinct absorption peaks were found at f_1, f_2, f_3, *and* f_4, respectively. (C) Simulated surface current density distributions on resonances. All four resonances originate from circulating currents in individual single-band resonators, which indicates that each absorption peak can be tuned independently in individual single-band resonators.

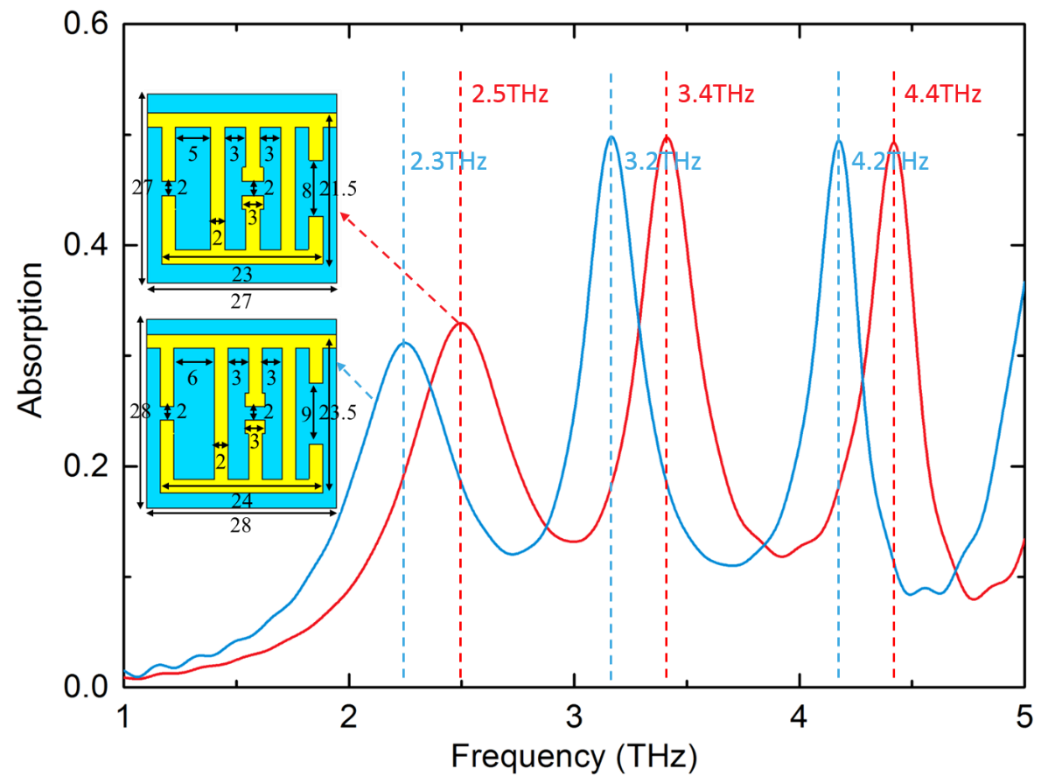

Figure S4. Tuning resonant frequencies by varying geometries of the multispectral metamaterial. Only LC resonances (which can be tuned by altering the dimension of the metamaterial) of the multispectral metamaterial are utilized in the design. Each of the LC resonances are derived from a single band SRRs and highly decoupled from the other adjacent ones. Thus all of the resonant frequencies of the multispectral can be tuned by changing geometries of the multispectral metamaterial.

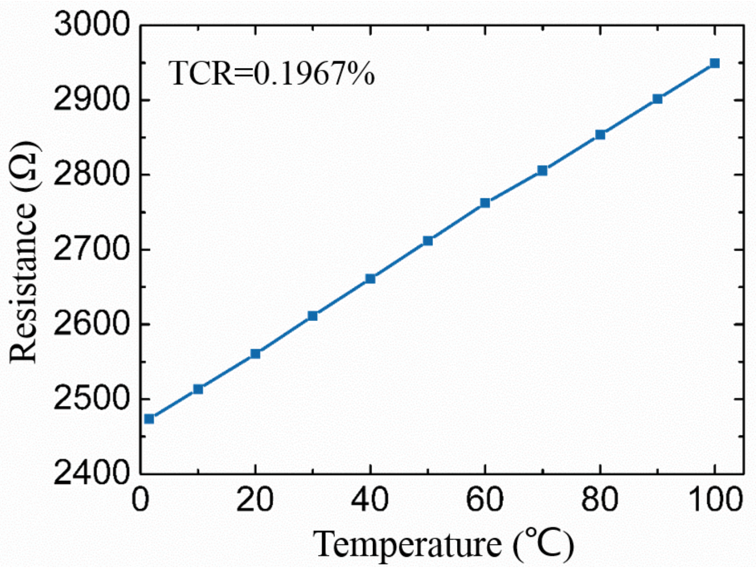

Figure S5. TCR of the multi-spectral THz FPA detector. The resistance of the device as a function of temperature. The temperature coefficient of resistance (TCR) of the device is 0.1967%.

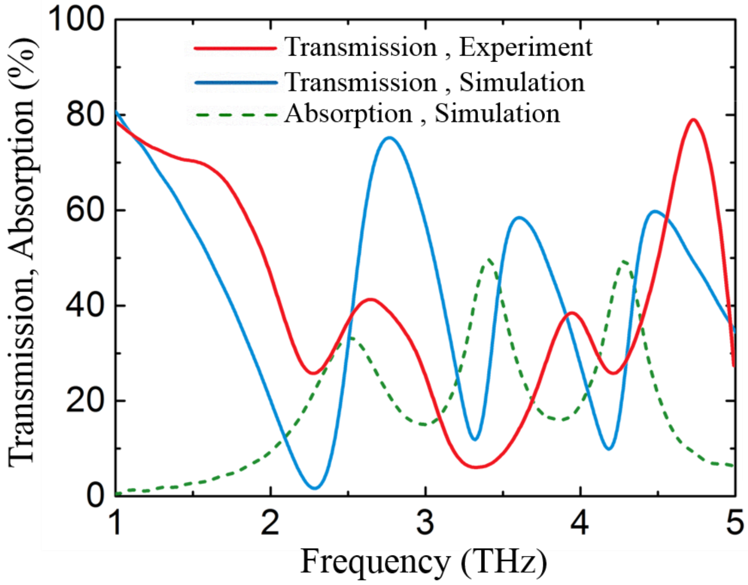

Figure S6. Spectral responses of multispectral metamaterials. Compared with the simulated transmission spectra, the characteristic valleys (or peaks) of the experimental and simulated results are in reasonable agreement. The resonances in experimental results were not as strong as the simulated one. Note that the characteristic valleys of the simulated transmission spectra do not overlap with the characteristic peaks of the simulated absorption spectra.

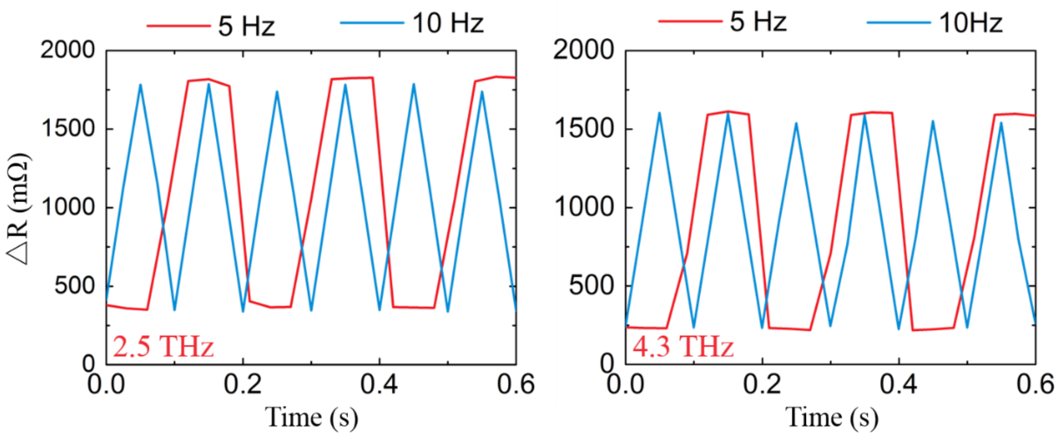

Figure S7. Temporal response of multi-spectral THz FPA detector. Temporal responses of the multi-spectral THz FPA detector were characterized by 2.5 THz (left panel) and 4.3 THz (right panel) QCL sources modulated at frequencies of 5 Hz (red) and 10 Hz (blue), respectively. The maximum operation frequency of the detector before the signal roll-off was 10 Hz with the current device on the two testing conditions, which can be further improved by optimization of device geometries of both the MM resonators and supporting cantilever legs (see below).

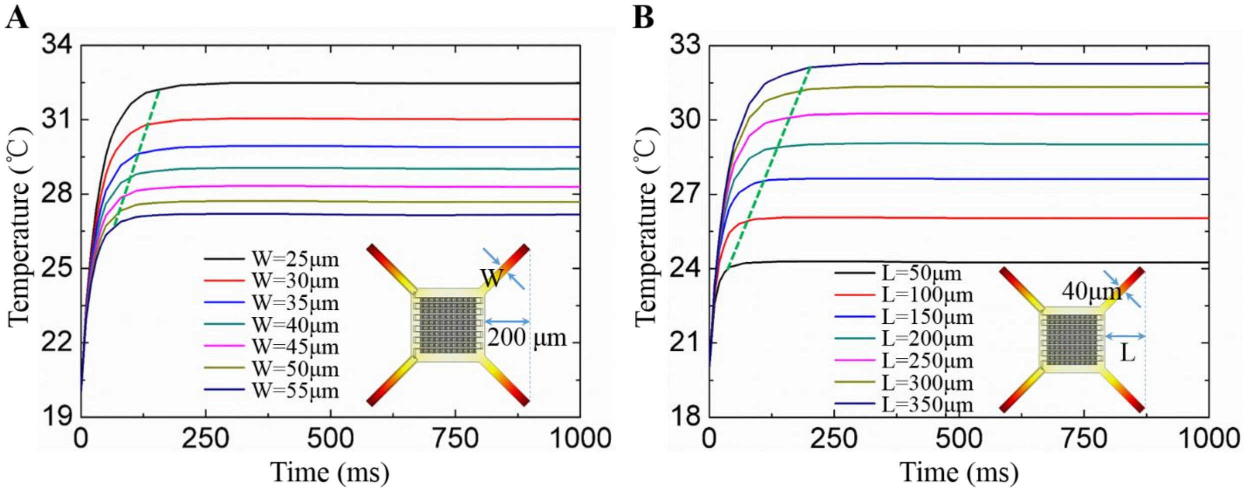

Figure S8. Compromise between responsivity and response time of the multi-spectral THz FPA detector. The increased temperature reflects the responsivity of the detector, and the time taken by the temperature to change from the original value to the steady value reflects the response time of the detector. The simulated transient thermal responses with different widths (A) and different lengths (B) of the thermal insulating silicon nitride cantilever legs. With increasing width or decreasing length, both the responsivity and the response time will decrease.

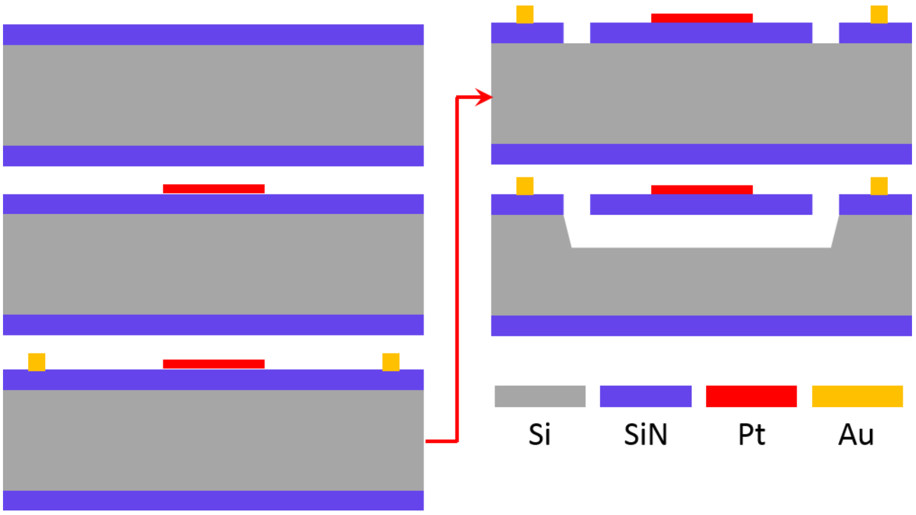

Figure S9. Fabrication process of THz FPA detector. Firstly, a low stress silicon nitride thin film with a thickness of 800 nm was deposited on a (100) silicon substrate. The Cr/Pt layer (10 nm /100 nm) defining the SRR array was then fabricated using a standard UV lithography followed by an ion beam etching process. Next, 30 nm / 300 nm Cr/Au defining the wire was performed using a standard liftoff process. Subsequently, the silicon nitride layer was patterned using the lithography and plasma etching processes to define the SRR array supporting membrane. Finally, the structures were released through TMAH wet etching of the silicon underneath the SRR array from the front side.

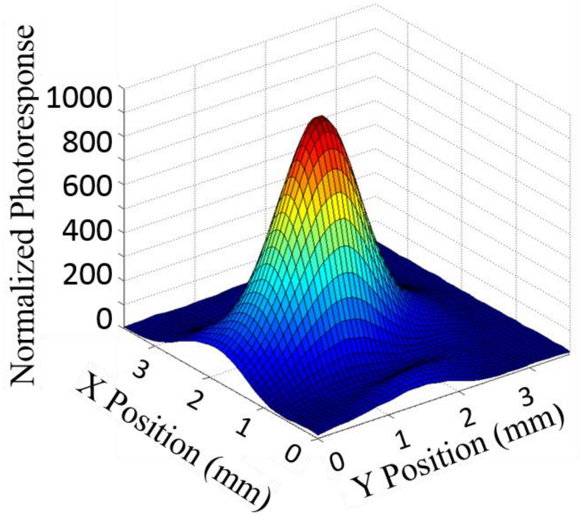

Figure S10. The intensity profile of the 3.4 THz QCL captured by a commercial THz camera. The result is obtained by using commercial THz camera (NEC IRV-T0831C) under the same measurement condition.

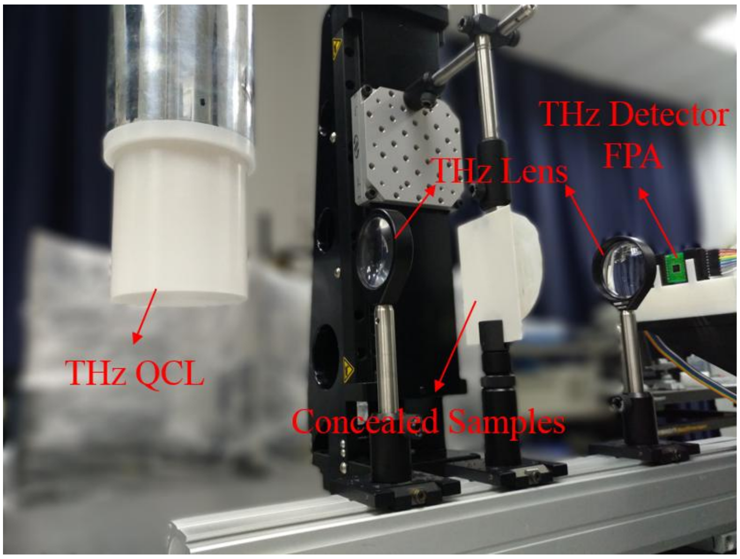

Figure S11. Photograph of a portion of the experimental setup for material identification based on T-ray imaging. The material to be identified was formed into tablets with PTFE powder at a concentration of 5 wt%. They were concealed by PTFE package with a thickness of 5 mm.

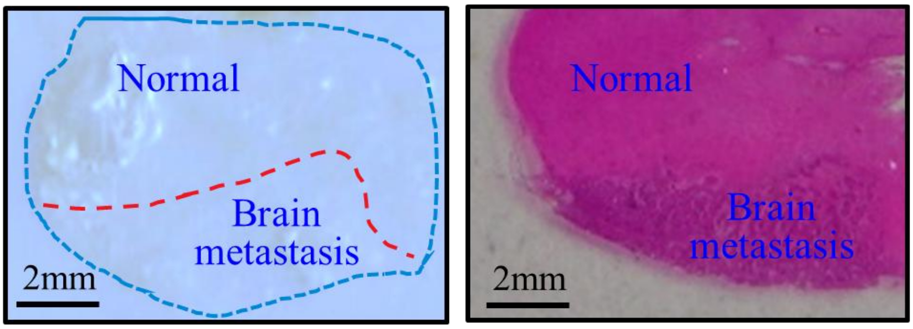

Figure S12. Hematoxylin and Eosin (H&E) stained image of the brain tissue. (Right) The bottom region stained deep purple with hematoxylin was the brain tumor region. (Left) Before the H&E staining, both healthy and cancerous tissues are optically transparent and cannot be distinguished visually. The boundary between normal tissue and brain tumor (in red dash line) is added based on H&E results for better illustration.

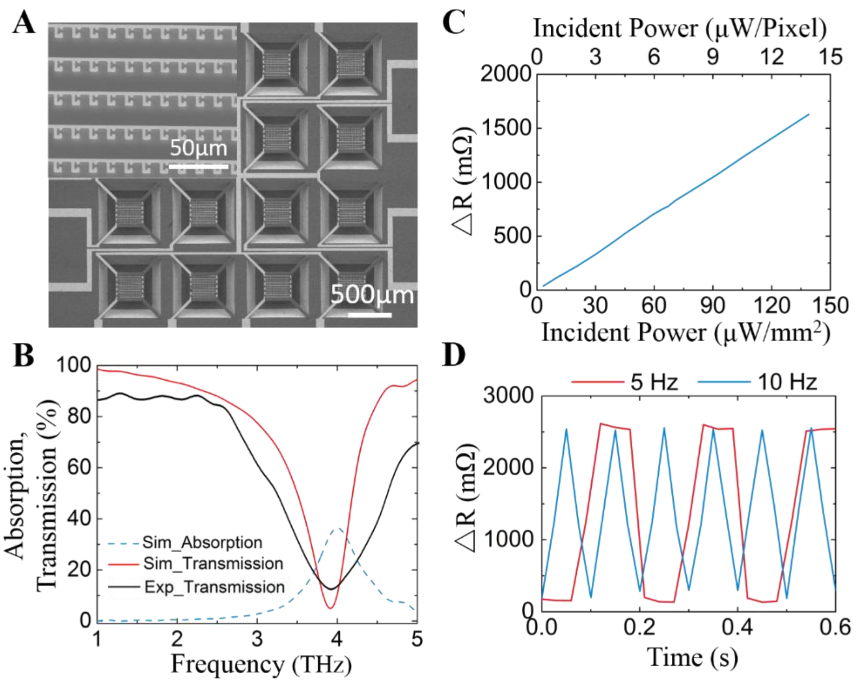

Fig. S13. Fabrication and characterization of the monolithic THz detector optimized for operating at 4.0 THz. (A) SEM images of a portion of the THz FPA detector. (B) Spectral responses. (C) The response of individual pixels characterized as a function of incident power irradiated by a 4.0 THz QCL source. (D) Temporal response of the monolithic THz FPA detector at 4.0 THz.

Table. S1. Performance comparison of THz detectors/imagers

Technology \ Performance	Frequency (THz)	NEP (pW/Hz$^{1/2}$)	Cooling	Array	Ref
Golay Cell	0.04-30	~ 140	Uncooled	No	[1]
Schottky Diode	0.15	~ 1.5	Uncooled	Yes	[2]
	0.28	~ 66	Uncooled	Yes	[3]
	0.86	~ 42	Uncooled	Yes	[4]
FET	0.20	~ 0.48	Uncooled	Yes	[5]
	0.82	~ 36	Uncooled	Yes	[6]
	0.14	~ 10	Uncooled	Yes	[7]
Bolometer	1 – 7	~ 100	Uncooled	Yes	[8]
	2.52	~ 45.7	Uncooled	Yes	[9]
	2.5	~ 108	Uncooled	Yes	[10]
THz QWP	4 – 7 THz	~ 5.2	3.5 – 10 K	Yes	[11]

[1] Tydex Golay detector data sheet, Tydex Corporation, Russia.

[2] J. L. Hesler, T. W. Crowe, "Responsivity and Noise Measurements of Zero-Bias Schottky Diode Detectors", presented at *Eighteenth International Symposium on Space Terahertz Technology,* 2007.

[3] R. Han, Y. Zhang, D. Coquillat, H. Videlier, W. Knap, E. Brown, K. O. Kenneth, *IEEE J. Solid-State Circuits* **2011**, *46*, 2602.

[4] R. Han, Y. Zhang, Y. Kim, D. Y. Kim, H. Shichijo, E. Afshari, K. O. Kenneth, *IEEE J. Solid-State Circuits* **2013**, *48*, 2296.

[5] Y. Kurita, G. Ducournau, D. Coquillat, A. Satou, K. Kobayashi, S. B. Tombet, Y. M. Meziani, V. V. Popov, W. Knap, T. Suemitsu, *Appl. Phys. Lett.* **2014**, *104*, 380.

[6] D. Y. Kim, S. Park, R. Han, K. O. Kenneth, *IEEE Trans. Terahertz Sci. Technol.* **2016**, *6*, 306.

[7] H. W. Hou, Z. Liu, J. H. Teng, T. Palacios, S. J. Chua, *Sci. Rep.* **2017**, *7*, 46664.

[8] NEC IR/V-T0831C Bolometer Specification, NEC Corporation., Tokyo, Japan, Sep 2014.

[9] J. Gou, J. Wang, X. Zheng, D. Gu, H. Yu, Y. Jiang, *RSC Adv.* **2015**, *5*, 84252.

[10] I. E. Carranza, J. P. Grant, J. Gough, D. Cumming, *IEEE J. Sel. Top. Quantum Electron.* **2017**, *23*, 1.

[11] Z. L. Fu, L. L. Gu, X. G. Guo, Z. Y. Tan, W. J. Wan, T. Zhou, D. X. Shao, R. Zhang, J. C. Cao, *Sci. Rep.* **2016**, *6*, 25383.
